# Roles of EvpP in *Edwardsiella piscicida-*Macrophage Interactions

**DOI:** 10.3389/fcimb.2020.00053

**Published:** 2020-02-14

**Authors:** Lei Qin, Xingqiang Wang, Yingli Gao, Keran Bi, Weixia Wang

**Affiliations:** ^1^Jiangsu Key Laboratory of Marine Biotechnology, College of Marine Life and Fisheries, Jiangsu Ocean University, Lianyungang, China; ^2^Co-Innovation Center of Jiangsu Marine Bio-industry Technology, Lianyungang, China

**Keywords:** *Edwardsiella piscicida*, EvpP, macrophage, RPS5, interaction

## Abstract

*Edwardsiella piscicida* is found to be an important facultative intracellular pathogen with a broad host range. These organisms can replicate and survive within host macrophages to escape from the subversion of the immune defense. *E. piscicida*-macrophage interaction is very important in determining the outcome of edwardsiellasis. As an effector protein of *E. piscicida* T6SS, EvpP has been determined to be a very important virulence factor for *E. piscicida*, although its precise role in *E. piscicida*-macrophage interactions is not yet clear. In this study, the roles of EvpP in *E. piscicida*-macrophage interactions were characterized. Here, we constructed the deletion mutants of *evp*P (Δ*evp*P) and complementation (Δ*evp*P-C) by the allelic exchange method. Compared to wild type strain (WT), Δ*evp*P was found to be attenuated for growth within macrophages. In line with this observation, we found its survival capacity was lower than WT under oxidative and acid stress *in vitro*, which simulate conditions encountered in host macrophages. Attenuation of Δ*evp*P also correlated with enhanced activation of macrophages, as reflected by augmented NO production in Δ*evp*P-treated macrophages. Moreover, compared to WT, Δ*evp*P induced markedly increased apoptosis of macrophages, characterized by increased Annexin V binding and the activation of cleaved caspase-3. These findings provided strong evidence that EvpP is involved in the process of *E. piscicida-*macrophage interactions and is required for its survival and replication in macrophages. Thus, we propose that EvpP might be an important factor that controlling the fate of *E. piscicida* inside macrophages. To further exploring the underlying mechanism of EvpP action, the cDNA library was constructed from *E. piscicida*-infected macrophages and a yeast two-hybrid screen was performed to search for cellular proteins interacting with EvpP. Ribosomal protein S5 (RPS5) was identified as a target of EvpP. Furthermore, the interaction was validated with co-immunoprecipitation assay. This result implies that the observed effect of EvpP on macrophages might be related to RPS5-mediated regulation, contributing to a better understanding of the mechanisms of EvpP involved in *E. piscicida*-macrophage interactions.

## Introduction

*Edwardsiella piscicida* is pathogen bacterium that could infect a wide range of hosts from fish to amphibians, bird, reptiles, and mammals including humans (Leung et al., 2012; Xu and Zhang, 2014), throughout the world. *E. piscicida* prefer to live inside host macrophages in order to replicate and escape from the subversion of the immune defense, eventually resulting in upgrading the local infection to a systemic infection (Padrós et al., 2006; Leung et al., 2012; Qin et al., 2014; Tan et al., 2019). The macrophage-pathogen interaction is thought to be vital in determining the outcome of infection (Bandin et al., 1993). However, the mechanism of how *E. piscicida* interacts with macrophages is mostly unknown, which is conductive to understand the strategies used by this pathogen to survive and replicated in macrophages.

The exact pathogenic mechanism of *E. piscicida* remains largely unknown, although a number of potential virulence factors have been proposed to be responsible for the pathogenesis of *E. piscicida a* (Leung et al., 2012). Among them, the type VI secretion systems (T6SS) has been determined as one of the most important components of virulence in *E. piscicida* (Zheng and Leung, 2007; Chakraborty et al., 2011). This system plays an essential role in allowing the bacteria to replicate intracellularly (Srinivasa Rao et al., 2010; Leung et al., 2012). The *E. piscicida* T6SS gene cluster encodes 16 open reading frames designated *evp*A to *evp*P. Several studies have demonstrated that EvpP is T6SS effector of *E. piscicida* (Zheng and Leung, 2007; Hu et al., 2014; Chen et al., 2017), although its exact role in pathogenesis of *E. piscicida* is not well understood. It has been reported that the transcription of *evp*P was regulated by two-component system EsrA-EseB (Wang et al., 2009), ferric uptake regulator (Fur) protein (Chakraborty et al., 2011), and H-NS protein (Zhang et al., 2014). EvpP could interact with EvpC inside the cytoplasm of bacteria (Zheng and Leung, 2007; Hu et al., 2014) and be translocated into the cytosol of infected cells from *E. piscicida* (Chen et al., 2017). In addition, EvpP was detected in outer membrane vesicles (OMVs) that naturally released from Gram-negative bacteria into the environment or as another means to deliver bacteria virulence factor into host cells (Park et al., 2011; Zhang et al., 2018). As an important T6SS effector, EvpP were found to be essential for virulence of *E. piscicida* (Hu et al., 2014). Deletion of *evp*P was found to decrease replication of *E. piscicida in vivo*, hemolytic activities and serum resistance, suggesting that EvpP may involve in *E. tarda* pathogenesis (Wang et al., 2009). Chen et al. (2017) reported that EvpP could prevent NLPR3 inflammasome activation by inhibiting Ca^2+^-Dependent MAPK-Jnk pathway in *E. piscicida*-infected macrophages. A recent study revealed the role of EvpP in promoting the pathogenesis of *E. piscicida* via inhibiting the phosphorylation of Jnk signaling, subsequently suppressing the caspy-inflammasome signaling cascades, contributing to the inhibition of neutrophils recruitment (Tan et al., 2019). In *E. ictalurid*, EvpP were demonstrated to be required for colonization of catfish ovary cell and increase apoptosis and necrosis in anterior kidney macrophages (Kalindamar et al., 2019). These reports increasingly reveal the importance of EvpP in *E. piscicida*-infection. Given the central importance of macrophages in the host immune response to *E. piscicida*, we inferred that EvpP may play an important role in the interactions between *E. piscicida* and macrophages, hence further exploring of the function of EvpP in *E. piscicida*-macrophage interaction is needed.

Although EvpP is involved in pathogenesis of *E. piscicida*, its detailed roles in *E. piscicida*-macrophages interactions have not been reported. In this study, we aimed to examine the functional importance of EvpP in the *E. piscicida-*macrophage interactions. Mouse macrophage lines including RAW264.7 cells have been usually used for study of *E. piscicida* pathogenesis because of its ease of culture, and phenotypic resemblance to primary macrophages (Okuda et al., 2006; Ishibe et al., 2009; Zhang et al., 2016; Chen et al., 2017; Qin et al., 2017; Gao et al., 2019). With this in mind, we select *E. piscicida*-infected RAW264.7 macrophages as cell model. Here, we demonstrated that EvpP is involved in the process of *E. piscicida-*macrophage interactions and is required for the its survival and replication in macrophages. Subsequent studies revealed that Ribosomal protein S5 (RPS5) was cellular proteins which could interact with EvpP. This work could increase our understanding of the diversity of EvpP functions involved in *E. piscicida*-macrophage interactions.

## Materials and Methods

### Bacterial Strains and Cells

*E. piscicida* strain QL-S (GenBank no. DQ233654) were stored at −70°C with 15% glycerol. Bacterial cultures were grown in TSA or TSB (Hangzhou Microbial Reagent Co., Ltd, China). RAW264.7 cells were purchased from the Cell Bank of Type Culture Collection of the Chinese Academy of Sciences and cultured in Dulbecco's modified Eagle's medium (DMEM) (GIBCO BRL, USA). The cells were passaged twice a week to keep an exponential growth.

### Construction of Deletion Mutants, Complementation

In-frame deletion mutants were constructed by the allelic exchange method, as described previously with some modifications (Luo et al., 2015). Briefly, the upstream and downstream regions of the target gene were PCR amplified from the genome of wild-type *E. piscicida* with primer MF1/MR1 and MF2/MR2, respectively (Table 1), fused together by overlapping extension PCR, and cloned into pLP12 suicide vector. The amplified plasmids pLP12-*evp*P were transformed into *E. coli* β2163 by electroporation. Then pLP12-*evp*P were mobilized into *E. piscicida* via biparental mating. Chloramphenicol were used for screening of insertional mutants and the vmt gene with L-arabinose for counter selection of deletion mutants. The deletion mutants Δ*evp*P were confirmed by PCR using primer MF1/MR2 and subsequent sequencing (Table 1). For complementation of Δ*evp*P, full-length *evp*P fragment was amplified from wild-type *E. piscicida* with using primer EXF/EXR (Table 1) and combined with linearized pBAD33 vector in the In-Fusion cloning. The resulting plasmid was transferred from *E. coli* β2163 into the Δ*evp*P by conjugation. The complementation *evp*P-C were confirmed by PCR using primer MCF-TF/MCF-TR and subsequent sequencing (Table 1).

**Table 1:** Primers used for construction of deletion mutants and complementation.

**Name**	**Sequence (5^**′**^-3^**′**^)**
MF1	CATGACGTCTAGA CGCTGACGCATCCATTATCGCAAC
MR1	GATATGCCCGACCGTCAGGTTTGGTCCAACATCGGACCATTCCAGCTC
MF2	GAGCTGGAATGGTCCGATGTTGGACCAAACCTGACGGTCGGGCATATC
MR2	TAGTCGTACTAGTTTGGCTGTCATCTCCGCTCAGGG
EXF	TGGGCTAGCGAATTCGAGCTAGGAGGAATTCACCATGTTGACTTTGAATTCCGAGCTG
EXR	TGCCTGCAGGTCGACTCTAGCTATTTCAATATTGAAAATTGGTGGCTAC
MCF-TF	CCATAAGATTAGCGGATCCTACCT
MCF-TR	CTTCTCTCATCCGCCAAAACAG

*The underlined sequences represent restriction enzyme sites used for cloning of PCR products*.

### Macrophage Survival Assay

The growth of tested strains under cultured conditions were measured in terms of OD at 600 nm_._ Macrophage survival assay was performed as described previously (Qin et al., 2017) with minor modifications. In brief, RAW264.7 cells were seeded in 24-well plates and incubated overnight before co-cultured with bacterial suspension at a multiplicity of infection (MOI) of 50 for 1 h. Cells were washed three times, and then incubated with 100 μg/mL gentamicin for 1 h to ensure that all extracellular bacteria were killed. The cells were washed with PBS and fresh DMEM with 10 μg/mL gentamicin were added. At 0, 8, 24, and 48 h post-infection, the cells were lysed with 1% Triton X-100 and the number of viable intracellular bacteria was calculated. Values were calculated from the mean of the bacterial population of two wells in triplicate experiments.

### Stress Resistance Assays

All the tested strains were cultured to the stationary growth phase in TSB (OD_600_ = 1.3). To assay the effects of acidification, H_2_O_2_, high-osmolarity stress and heat treatment, the cells were incubated at pH 3.0 for 15 min, in the presence of 440 mM H_2_O_2_ for 40 min, in the presence of 1.5 M NaCl for 20 min, and at 50°C for 60 min, respectively. The bacteria were serially diluted and spread on TSA for CFU determination. Each experiment was performed in triplicate.

### Nitric Oxide (NO) Determination

The evaluation of NO production was performed by the Greiss assay. Briefly, cells in 24-well plates were incubated with bacterial suspension at a multiplicity of infection (MOI) of 50. At 12 and 24 h post-infection, the level of NO was determined by measuring the amount of nitrite in the culture medium using a NO detection Kit (Nanjing Jiancheng bioengineering Institute, China). The optical density of each well was determined using a microplate reader with an emission wave length at 550 nm.

### Flow Cytometry

Infections were conducted as described above for the macrophage survival assay. At the end the 24 and 48 h post-incubation, cells were collected and resuspended in binding buffer at density of 1 × 10^6^ cells/mL. The cell suspension was stained with 5 μL Annexin V-FITC and 10 μL propidium iodide (Annexin V-FITC Apoptosis Detection Kit, Sigma Aldrich, USA) for 15 min at room temperatures in the dark. Quantification of apoptotic cells was analysis by flow cytometry (Accuri C6, BD). The percentage of apoptotic cells was defined as the sum of early and late apoptotic cells.

### Western Blot Analysis

Infection experiment were conducted as described above for the apoptosis assay. Following infection, cells were lysed for western blot analysis. Briefly, equal amounts of protein from samples were separated on a 10% SDS-PAGE gel and transferred to a polyvinylidene difluoride membrane. GAPDH antibody (Abcam, USA) was used as the loading control. After being blocked with 5% BSA, the membrane was incubated overnight with caspase-3 antibody (Abcam, USA). Following extensive washing, the membrane was then incubated with appropriate secondary antibodies for 2 h at room temperature. The proteins were visualized with Western Lightning™ Chemiluminescence Reagent Plus kit (Perkin Elmer, USA). The relative protein expression intensities were quantified by densitometry using Labworks™ Analysis Software (UVP, USA). Each reaction was performed in triplicate.

### Construction of Yeast Two-Hybrid cDNA Library

The RAW264.7 cells were treated as described above for the intracellular replication experiment. At the end of 5 h post-incubation, total RNA was isolated for cDNA library construction. Make Your Own “Mate & Plate™” Library system (Clontech, Japan) was used to establish cDNA library as described by Cai et al. (2016).

### Vector Construction

The full-length sequence of *evp*P was synthesized and cloned into pGBKT7 to generate pGBKT7-*evp*P as a bait plasmid. In addition, *evp*P was cloned into pcDNA3.1 (+) with a Myc-tag to generate Myc-*evp*P. The RPS5 gene was amplified and inserted into pcDNA3.1 (+) with a HA-tag to create HA-RPS5. The validity of all vectors was verified by enzymatic digestion and DNA sequencing.

### Bait Plasmid Expression, Auto-Activation and Toxicity Tests

The pGBKT7-*evp*P plasmid and empty pGBKT7 vector were transformed into the yeast strain Y2HGold using theYeast maker™ Yeast Transformation System 2 kit according to the manufacturer's manual (Clontech, Japan), respectively. Total proteins were detected using c-myc tag mouse McAb (Tianjin Saierbio, China) to detect *evp*P expression as described previously (Lai and Lau, 2017). The culture with recombinant pGBKT7-*evp*P bait were plated on SD/-Trp, SD/-Trp/X and SD/-Trp/X/A plates. Based on white colonies on SD/-Trp and SD/-Trp/X plates and absence of colony growth on SD/-Trp/X/A plates, the pGBKT7- *evp*P bait was confirmed to have no auto-activation. Moreover, similar colonies size between pGBKT7- *evp*P bait and pGBKT7 plasmids could confirm it has no toxicity.

### Yeast Two-Hybrid Screening

Following elimination of autoactivation and toxicity of EvpP, Y2HGold transformed with pGBKT7- *evp*P was mated with Y187 containing the cDNA library. The heterozygotes were grown on SD/-Trp-Leu (DDO) plates. The clones were then transferred to SD/-Leu-Trp-His-Ade/X/A (QDO/X/A) plates three times. The blue colonies on QDO/X/A agar plates were confirmed as positive hits and the corresponding prey plasmids were sequenced and BLAST-aligned using NCBI. To eliminate false positive hits, prey plasmids was co-transformed with pGBKT7-*evp*P into Y2HGold cells and the co-transformants were grown on QDO/X/A plates. Co-transformation with BD-p53/AD-T and BD-Lam/AD-T served as positive and negative controls, respectively.

### Co-immunoprecipitation Assay

To validate the EvpP-Rps5 interaction discovered using the two-hybrid system in yeast, we performed coimmunoprecipitation (Co-IP) experiments in 293T cell line. Cells were grown until 80% confluence and co-transfected with 5 μg Myc- *evp*P and 5 μg HA- Rps5 (Co-transfection with Myc and HA-Rps5 or Myc- *evp*P and HA as a control). At 48 h post-incubation, the cells were lysed and the 80% of supernatant was subjected to co-IP experiments. The lysate was immunoprecipitated with anti-HA antibody (normal IgG as a control) followed by western blot analysis. The inverse coimmunoprecipitation experiment was also performed with anti-Myc antibody (normal IgG as a control). Briefly, mouse anti-HA (Sigma Aldrich, USA), mouse anti-myc (Sigma Aldrich, USA) or normal mouse IgG control antibody (Santa Cruz, USA) were separately added into the protein lysate, and incubated overnight at 4°C, followed by adding 30 μL Protein A/G magnetic beads (Thermo Scientific, USA) and incubating overnight at 4°C. Subsequently, the beads were gently washed by centrifugation 3 times and subjected to western blot analysis with rabbit anti-Myc (Sigma Aldrich, USA) and rabbit anti-HA antibody (Sigma Aldrich, USA), respectively. Input assays were performed using the remaining 20% of the supernatant for analysis of HA-Rps5 and Myc- *evp*P protein expression. All experiments were repeated at least three times independently.

### Statistical Analyses

All data are expressed as the mean ± standard deviation (SD) and statistically analyzed using one-way ANOVA followed by Tukey's Multiple Comparison test. *P* < 0.05 was considered statistically significant throughout the study.

## Results

### Δ*evp*P Is Attenuated in Macrophages

**Figure 1:**
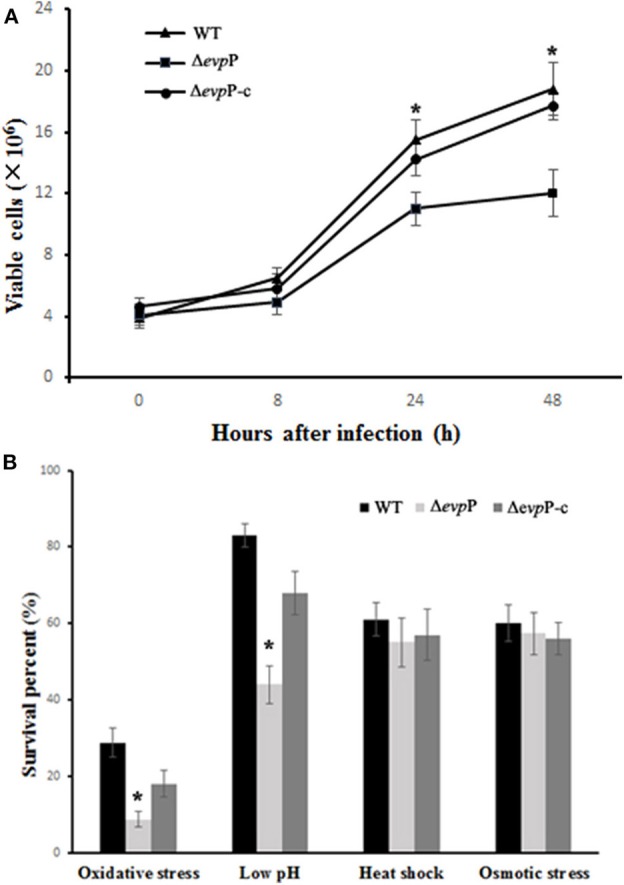
EvpP involved in intracellular survival. **(A)** Survival in macrophage cells. The numbers of recovered viable bacteria are shown as the means ± SD for three trials in duplicate wells. **(B)** Survival under stress conditions. The result was expressed as survival percent relative to the untreated controls. *P < 0.05.

To define the role of EvpP in the intracellular survival of *E. piscicida, evp*P deletion mutant (Δ*evp*P) and complementation Δ*evp*P (Δ*evp*P-C) were constructed. We compared the growth of these three strains under cultured conditions to exam whether the deletion of the *evp*P affected the growth of *E. piscicida in vitro*. The growth curves demonstrated that there was no notable difference in the growth rate of Δ*evp*P and WT (Figure S1). The survival capacities of Δ*evp*P were then evaluated under *in vitro* stress conditions and in macrophages. The results showed that the number of recovered Δ*evp*P was significantly lower than that of WT. Nevertheless, low-level replication of Δ*evp*P was still detected in macrophages during infection (Figure 1A). Morever, we found that the survival capacity of Δ*evp*P was significantly reduced under oxidative and acid stress which simulate *in vivo* conditions that *E. piscicida* encounters in host macrophages (Figure 1B). These results suggest that WT could survive and replicate within macrophages, while Δ*evp*P has an impaired ability to support its sustaining proliferation in macrophages.

### Δ*evp*P Induced More NO Production in Macrophages

**Figure 2:**
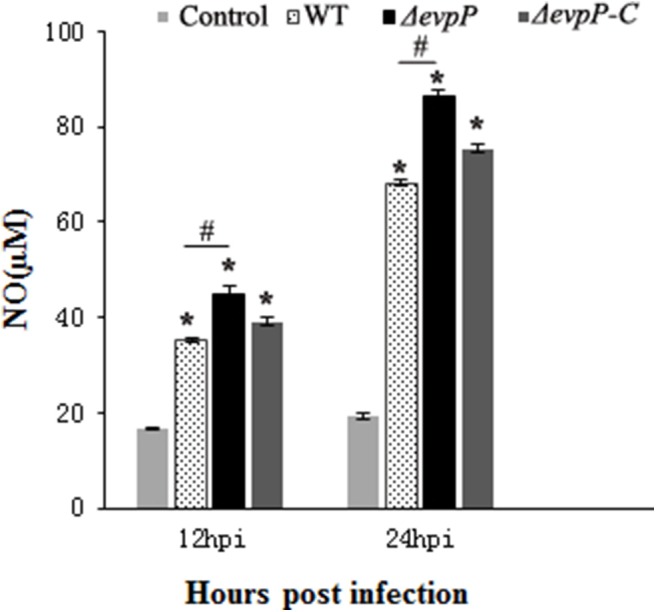
Effect of EvpP on NO production in RAW264.7 cells. NO was determined by measuring the amount of nitrite in the culture media using Griess reagents. *P < 0.05; ^#^*P* < 0.05.

The roles of EvpP involved in macrophage activation was examined by measuring NO production of macrophages following infected with bacterial suspension. The results showed that, compared to treatment with WT, treatment with Δ*evp*P significantly increased NO release of the macrophages (Figure 2). These results imply that EvpP could inhibit the activation of macrophages, resulting in a low NO level in *E. piscicida*-infected cells.

### Δ*evp*P Triggered Increased Apoptosis of Macrophages

**Figure 3:**
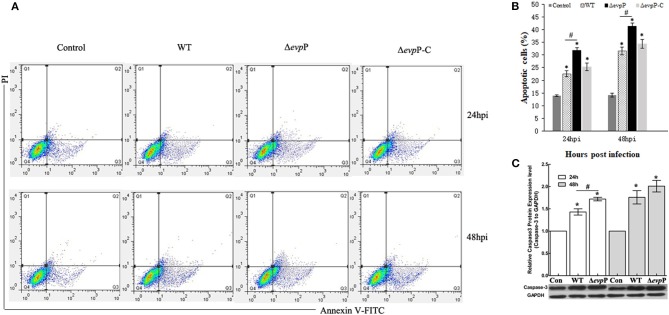
Δ*evp*P involved in apoptosis of macrophages. **(A)** Annexin V-FITC/PI double staining assay. Cells in the lower left quadrant are viable, those in the lower right quadrant are early apoptotic and those in the upper right quadrants are late apoptotic. **(B)** The degree of apoptotic cell death was quantified. The sum of early and late apoptotic cells was calculated as apoptotic cells. Data obtained from three separate experiments. **(C)** Cleaved caspase-3 examined by western blot analysis. The relative quantity of caspase-3 protein expression was normalized with relative to the level of GAPDH. Data represent the mean ± SD of three independent experiments. *P < 0.05; ^#^*P* < 0.05.

The effect of EvpP on apoptosis of macrophages was verified by flow cytometry analysis. The result demonstrated that Δ*evp*P induced more increased apoptosis rate of macrophages compared to that of WT (Figures 3A,B). In addition, we found that Δ*evp*p induced more early apoptosis than late apoptosis. To confirm these results, we measured the expressions of active caspase-3 in RAW264.7 cells at 24 and 48 h post-infection by western blotting. As demonstrated in Figure 3C, compared with WT, more increased level of caspase-3 were detected in Δ*evp*P-infected macrophages in parallel with increased apoptosis observed in flow cytometry assay.

### Construction of Yeast Two-Hybrid cDNA Library

**Figure 4:**
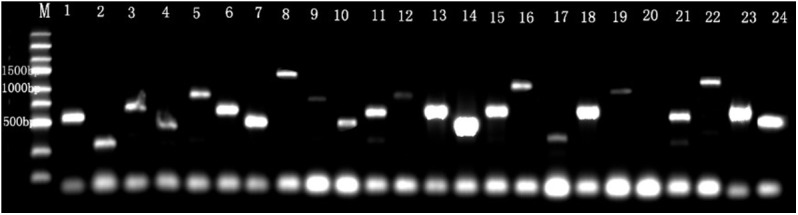
The identification of the inserts size in cDNA library. M, Marker. Line 1–24 were 24 recombinant individual colonies, which were randomly picked and amplified by PCR.

Detection of the library indicated that the transformation efficiency of the library was 2.2 × 10^6^ cfu/μg of vector and the titer of cDNA library was 3.7 × 10^7^ cfu/mL. Twenty-four colonies were randomly picked to run PCR using the AD vector universal primers, showing that the inserted DNA fragment size was range from 0.4 to 1.5 kb (Figure 4). These results demonstrate that the library meets the requirements of the standard cDNA library.

### Screening of Cellular Proteins That Interact With EvpP

**Figure 5:**
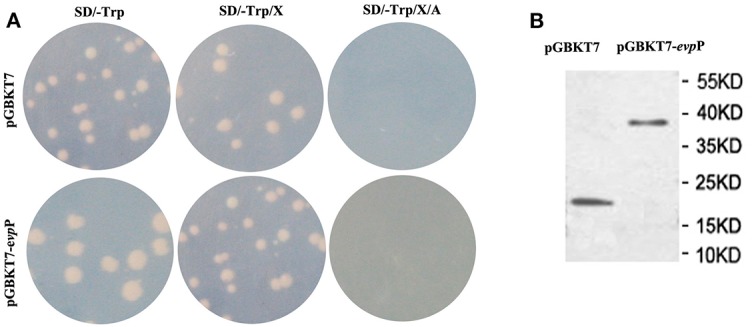
Expression and auto-activation tests for pGBKT7-*evp*P. **(A)** Analysis of auto-activation activity of pGBKT7-*evp*P plasmid in Y2HGold cells. The pGBKT7-*evp*P and pGBKT7 plasmids were transformed to Y2HGold cells and the transformants were grown on SD, SD/X, and SD/X/A agar plates, respectively. **(B)** Western blot detection of pGBKT7-*evp*P and pGBKT7 expression in Y2HGold cells.

To identify the interactive proteins of EvpP, the yeast strain Y2HGold containing BD- *evp*P was mated with strain Y187 containing cDNA library from *E. piscicida*-infected macrophage. The auto-activation test demonstrated that pGBKT7- *evp*P and pGBKT7 alone did not autonomously activate the reporter gene (Figure 5A). Also, toxicity test confirmed that *evp*P bait has no toxicity to the Y2HGold strain. The auto-activation and toxicity test indicated that the constructs were suitable for Y2H screening. In addition, total proteins of the Y2HGold transformed with pGBKT7- *evp*P and pGBKT7 plasmids were detected by western blotting. As shown in Figure 5B, the molecular weight of pGBKT7- *evp*P and pGBKT7 were 38 and 20 kDa, respectively, suggesting that EvpP could be expressed in Y2HGold strain.

**Figure 6:**
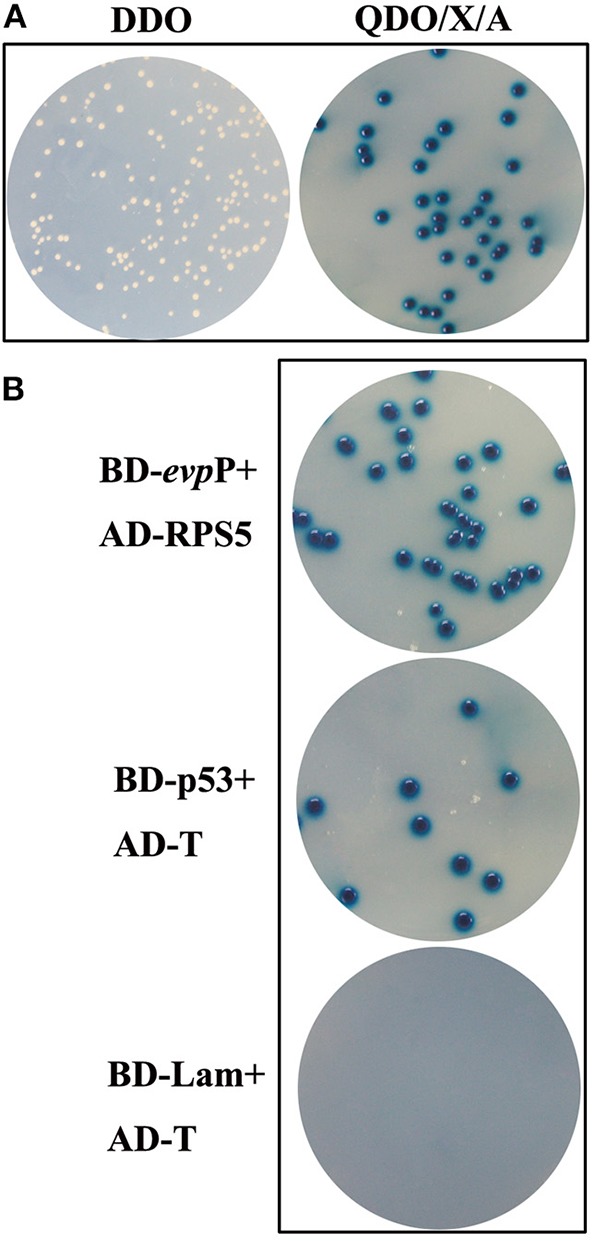
Yeast two-hybrid screening. **(A)** Colonies on DDO and QDO/X/A agar plates. Blue colonies on QDO/X/A agar plates were confirmed as positive hits. **(B)** Confirmation of putative hits. AD-RPS5 was co-transformed with BD-*evp*P into Y2HGold cells. The co-transformations were plated on QDO/X/A plates. Co-transformations with BD-p53/AD-T and BD-Lam/AD-T served as positive and negative controls, respectively.

Following screening on higher stringency QDO/X/A plates, ten blue colonies were finally obtained, which were likely to be positive hit (Figure 6A). Sequence analysis of the positive prey plasmid resulting in 5 candidate genes. Among them, 1 gene was finally selected, which displayed 98% similarity with *Mus musculus* ribosomal protein S5(RPS5) (Accession no.: NM_009095.2). Other 4 genes were ruled out for its very low sequence homology. To confirm the specificity of the interaction, prey plasmids were co-transformed with pGBKT7-*evp*P into Y2HGold cells, resulting in blue colonies growth on QDO/X/A plates. Co-transformations with BD-p53/AD-T and BD-Lam/AD-T as positive, and negative controls, respectively, indicated that the experiments were successful (Figure 6B).

### Interaction Between EvpP and Rps5 Was Confirmed by Co-immunoprecipitation Assay

**Figure 7:**
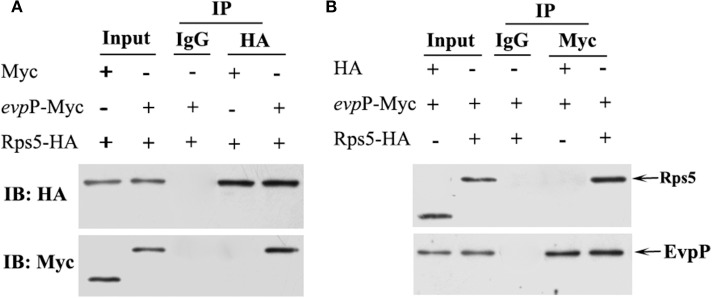
Co-IP analysis of EvpP and Rps5. 293T cells were co-transfected with Myc-*evp*P and HA-Rps. The 20% of the cell lysates were subjected to the input assay to assess Myc-*evp*P and HA- Rps5 protein levels. **(A)** The lysates were subjected to IP assay with mouse anti-HA or IgG control antibody, and the precipitated proteins were analyzed by western blot with rabbit anti-HA and anti-Myc, respectively. 293T cells were also co-transfected with Myc and HA-Rps5 as a control. **(B)** The IP assay was performed with mouse anti-Myc or IgG control antibody, followed by detection of the precipitated proteins by western blot with rabbit anti-HA and anti-Myc, respectively. 293T cells were co-transfected with Myc-*evp*P and HA as a control.

Co-IP assays were used to confirm the interaction between EvpP and Rps5. In the HA- Rps5 immunoprecipitation, we searched for EvpP through western blotting. Aiming to confirm the interaction, we also immunoprecipitated EvpP and searched for Rps5 through western blotting. The result of co-IP followed by western blotting is shown in Figure 7. Both EvpP and Rps5 could be detected in cell lysates. When Rps5 was immunoprecipitated, more EvpP were coimmunoprecipitated, and no Myc were detected from Myc and HA-Rps5 co-transfected cells, presenting additional evidence of a EvpP:Rps5 interaction inside cells (Figure 7A). Furthermore, the inverse coimmunoprecipitation experiment was performed, and the immunoprecipitated EvpP were able to bring Rps5, and no HA were detected from Myc-EvpP and HA co-transfected cells, reinforcing the EvpP:Rps5 interaction (Figure 7B).

## Discussion

To define the roles of EvpP in *E. piscicida*-macrophage interactions, we constructed an *evp*P-defective *E. piscicida* mutant and compared its performance with that of the wild type in terms of intracellular survival, stress resistance, NO generation and induced apoptosis of macrophages. In our study, Δ*evp*P was found to be attenuated for growth within macrophages, whereas WT and Δ*evp*P-C were able to establish a stable infection, suggesting the importance of EvpP for *E. piscicida* survival in macrophages. Intracellular pathogen could invade and replicate in macrophages, where encounters a variety of environmental stress, including low pH, oxidative stresses, heat shock, etc. (Fontan et al., 2008). To further characterize the Δ*evp*P, we performed stress resistance assay *in vitro*, using conditions that mimic the macrophage environment. The data showed that mutation of *evp*P renders the cells more sensitive to oxidative and acid stress, resulting in weakening the ability of *E. piscicida* to resist against the stress of macrophages. This result further corroborated our observations that intracellular survival of Δ*evp*P could be impaired in macrophages, suggesting that EvpP is required for the survival and replication of *E. piscicida* in macrophages. In addition, Δ*evp*P were found to cause enhanced activation of macrophages, as reflected by augmented NO production. Previous studies have focused on the role of NO as the macrophage microbicidal mediator, implicating NO as an essential element in intracellular pathogen killing (Darrah et al., 2000). It is well established that macrophages are activated to produce NO when exposed to *E. piscicida* (Ishibe et al., 2009; Qin et al., 2017). In contrast with WT, Δ*evp*P induced more NO production in macrophages, probably accounting in part for the low-level intracellular replication in cells, indicating that EvpP might exert an inhibitory effect on NO release of macrophages.

There is growing evidence that apoptosis could give help to the host immune response against the intracellular bacteria, revealing the importance of apoptosis in host-pathogen interactions (Gao and Kwaik, 2000). Apoptosis were beneficial to intracellular pathogen elimination by removing the favorable intracellular niche for survival. Pathogen and its host cells have developed a complex interaction in which the pathogen could strive to survive and replicate (Vaux and Häcker, 1995). Thus, it is not surprising that *E. piscicida* could evolve mechanisms to inhibit macrophages apoptosis. Anti-apoptosis has been viewed as an attempt by the intracellular pathogen to reduce apoptosis to allow enough time for a change in phenotype to be complete before escaping from the apoptotic cells (Bermudez et al., 1997). *E. piscicida* was demonstrated to induce an anti-apoptotic effect on murine macrophages through the up-regulation of anti-apoptotic genes for the intracellular survival (Okuda et al., 2006). In our study, flow cytometry analysis showed that, compared to WT strain, Δ*evp*P induced markedly increased apoptosis rate of macrophages. As a critical executioner of apoptosis, increased caspase-3 activity was detected in Δ*evp*P-infected macrophages, contributing to confirming enhanced apoptosis triggered by Δ*evp*P. These results imply that EvpP participate in anti-apoptotic mechanisms which may be an important strategy employed by *E. piscicida*, thereby block macrophages apoptosis to benefit their survival. However, the exact mechanisms of EvpP involved in anti-apoptosis require further study.

To further exploring the underlying mechanism of EvpP exerting effect on macrophages, the cDNA library was constructed from *E. piscicida*-infected macrophages and the Y2H system was adopted to search for host proteins interacting with EvpP, showing that Ribosomal protein S5 (RPS5) is a target of EvpP. Furthermore, the interaction was validated with co-immunoprecipitation assay, indicating the reliability of the Y2H approach. These results imply that EvpP might exert an influence on host macrophages by means of interaction with RPS5. RPS5, as an important component of ribosomes, forms part of the exit site on the small ribosomal subunit and cross-links to the exit-site tRNA (Matragkou et al., 2009). RPS5 is known for its involvement in ribosome biogenesis function, however its functions remain largely unknown. The non-ribosomal functions of ribosomal proteins have recently attracted worldwide attention. The increasingly accumulated evidence suggests that ribosomal proteins has extra-ribosomal functions associated with cell differentiation, apoptosis, regulation of signal pathway, and DNA repair (LindstrÖm, 2009; Warner and Mcintosh, 2009). RPS5 was also found to participate in extra-ribosomal activities such as cell differentiation (Matragkou et al., 2008; Vizirianakis et al., 2015), intracellular trafficking (Matragkou et al., 2009), and hepatic fibrosis (Xu et al., 2016).

It is worthwhile to note that RPS5 has been demonstrated to possess important other extra-ribosomal functions including apoptosis and signal transduction (Vizirianakis et al., 1999; Li et al., 2006; Xu et al., 2016; Zhi et al., 2018). The caspase-recruitment domain (CARD) is found in RPS5, which is known to play an important role in apoptosis and inflammation as an essential protein–protein interaction domains (Wilson et al., 1985; Li et al., 2006). Vaughn et al. (1999) reported that CARD of Apaf-1 could bind to caspase-9 to trigger a proteolytic cascade, resulting in apoptotic cell death. Many CARD-containing proteins have been considered as important players in the innate immune response (Thiagarajan et al., 2006). Apoptosis assay in our study demonstrated that EvpP could suppress apoptosis of macrophages. Therefore, we may speculate that it is most likely that interaction of EvpP with RPS5 negatively regulate apoptosis-associated pathway, thus resulting in reduced apoptosis of macrophages to aid their survival. Detailed mechanism of how EvpP regulate apoptosis by means of interaction with RPS5 to remain to be elucidated, and the function of RPS5 in the *E. piscicida*-induced apoptosis warrant further investigation. In addition to its role involved in apoptosis, RPS5 has been implicated in performing function involved in signal transduction. RPS5 could participate in Akt signaling pathway and were found to reduces Akt Phosphorylation (Xu et al., 2014). Further study showed that RPS5 knockdown substantially promoted LPS-induced IκB phosphorylation and degradation and enhanced LPS-induced ERK, JNK, and p38 phosphorylation (Xu et al., 2016). Moreover, Zhi et al. (2018) observed the regulation effect of RPS5 on NF-kB, MAPK pathways and confirmed the important role of RPS5 in intracellular cell signaling transduction. These available evidences allow us to presume that the effects of EvpP on macrophages is related to RPS5-mediated signal transduction. Our study demonstrated that EvpP could inhibit the release of NO from *E. piscicida*-infected macrophages. It is well-known that increasing production of NO is closely related to activation of NF-κB signaling pathway (Akira and Takeda, 2004; Darieva et al., 2004). Thus, we would like to presume that the inhibitory effect of EvpP on NO production of macrophages might be relate to RPS5-mediated signal pathways, ultimately resulting in a reduced release of NO. Further work is needed to test this hypothesis.

Interestingly, EvpP was reported to prevent the NLPR3 inflammasome activation-mediated pyroptosis by inhibiting intracellular Ca^2+^-dependent MAPK-Jnk pathway in *E. piscicida*-infected macrophages (Chen et al., 2017). However, the mechanism of how EvpP regulates intracellular Ca^2+^ signaling is still unclear. Perhaps, the screen of target proteins interacting with EvpP in our study will contribute to explaining how EvpP regulates intracellular Ca^2+^ signaling. Given the prominent role of RPS5 in cell signaling transduction, our findings imply that effects of EvpP on intracellular Ca^2+^ signaling in macrophages might be achieved by PRS5-mediated signal pathway, although the precise mechanism of its linkage to RPS5 is remains unclear. Combined with our research results, the finding of pyroptosis in *E. piscicida*-infected macrophages revealed that EvpP might offer a double protective function for *E. piscicida* survival in macrophages via its anti-pyroptosis and anti-apoptosis properties. Nevertheless, in present study, it is noteworthy that more early apoptosis could be observed than late apoptosis in *E. piscicida*-infected macrophages, implying that cell death by apoptosis might play a significant role in *E. piscicida*-macrophage interactions. Although EvpP have been demonstrated to exert inhibitory effect on apoptosis and pyroptosis in *E. piscicida*-infected macrophages, for *E. ictalurid*, EvpP were shown to increase apoptosis and necrosis in anterior kidney macrophages (Kalindamar et al., 2019). Additionally, in contrast to *E. piscicida*, deletion of *evp*P were not found to affect the virulence of *E. ictalurid* significantly in that study. These contrasting observations remain to be explained, implicating that EvpP might play diverse roles in different pathogens confronting different cellular hosts.

Taken together, our study uncovered a significant role of EvpP associated with *E. piscicida-*macrophages interaction. Our results indicate that EvpP of *E. piscicida* is required for its survival and replication in macrophages. Furthermore, we revealed that EvpP interacts with RPS5 in infected macrophages providing insight into the function of EvpP. However, further investigation relating to the EvpP-RPS5 interaction and the function of RPS5 in *E. piscicida* infection will be performed in a future study.

## Data Availability Statement

The datasets generated for this study are available on request to the corresponding author.

## Author Contributions

LQ conceived the planning of the study, design of experiments, mentored the researchers, wrote the paper, and contributed to some of the experiments. XW, YG, and WW conducted the experiments. KB contributed the technical assistance.

### Conflict of Interest

The authors declare that the research was conducted in the absence of any commercial or financial relationships that could be construed as a potential conflict of interest.
