# Supplementary material for: Roles of EvpP in Edwardsiella piscicida-Macrophage Interactions
Source: Front Cell Infect Microbiol. 2020 Feb 14;10:53. doi: 10.3389/fcimb.2020.00053 (PMC7033576; doi:10.3389/fcimb.2020.00053)
Supplement: Supplementary file 1 [file Data_Sheet_1.docx]

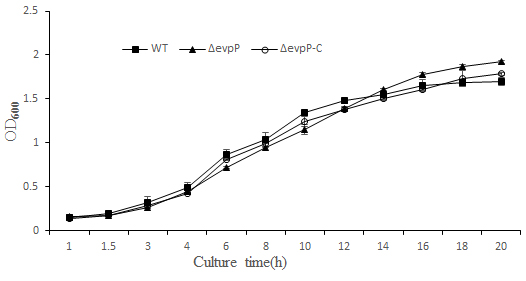


**FIGURE S1** Comparison of cell growth in WT, Δ*evp*P and Δ*evp*P -C. All experiments were performed in triplicate.
